# Prevalence and Genetic Diversity of HAV and HBV Viruses among Jaundice Patients at Coast General Hospital, Mombasa County, Kenya

**DOI:** 10.21315/mjms2021.28.3.5

**Published:** 2021-06-30

**Authors:** Gordon Ochieng’ Kasera, John M Maingi, Omondi Kevin Onyango, Anthony Kebira Nyamache

**Affiliations:** Department of Biochemistry, Biotechnology and Microbiology, Kenyatta University, Nairobi, Kenya

**Keywords:** prevalence, genetic diversity, HAV, HBV, jaundice

## Abstract

**Background:**

Hepatitis A and B causes morbidity and mortality among patients. This study determined the proportion of hepatitis A, B viruses (HAV, HBV) and genetic diversity of HBV among jaundice patients at the Coast General Hospital, Mombasa County, Kenya.

**Methods:**

A cross-sectional study was conducted among 222 patients; recruited and screened for hepatitis B surface antigen (HBsAg) and anti-HAV IgM. Viral deoxyribonucleic acid (DNA) was extracted from positive samples; partial hepatitis B virus-*pol* (HBV-*pol*) gene amplified, directly sequenced and generated sequences phylogenetically analysed using MEGA X software. Demographic characteristics were compared in relation to HBV infection using Chi-square.

**Results:**

Forty-seven (21.2%) out of the 222 patients tested positive for HBV while no HAV was detected. Among those infected, *n* = 8 (3.6%) were females and *n* = 39 (17.6%) males. Forty-five samples amplified and sequenced successfully. However, two samples failed to amplify. Phylogenetic analysis revealed HBV A1 genotype [*n* = 35 (74.5%)] was most predominant. A3, B and C2 genotypes each occurred [*n* = 1 (0.02%)]. This study revealed co-existence of HBV A3, B and C2 genotypes that have not yet been detected in this region.

**Conclusion:**

HBV A1 genotype remains the predominant genotypes in this region. The detected HBV prevalence indicates possible high transmission with possibility of increasing trends of HBV genotypes based on revelation of existence of new genotypes in this region.

## Introduction

Viral hepatitis is a great public health problem world over with hepatitis B being the most predominant. Hepatitis A is asymptomatic at early stages of infection (1, 2) and is majorly transmitted through faecal-oral route due to poor water and food hygiene (3). However, in hepatitis B virus (HBV), the infection is mostly through contact with infected blood and body fluids, hence making it one of the most highly infectious agents (4, 5). HBV being endemic in sub-Saharan Africa (6), its prevalence could range from 2% to 20% (7). Endemicity of these infections could therefore be categorised as either low (< 2.0%), low intermediate (2.0%–4.0%), high intermediate (5.0%–7.0%) or high (≥ 8.0%) endemicity (8).

For instance, in Kenya, the HBV prevalence is low intermediate endemic (8) while those of hepatitis A virus (HAV) is high intermediate which ranges from 2.0% to 9.2% (9, 10). HBV has A-H genotypes with HBV A, D, D/E genotypes and A1 and D6 sub-genotypes have been detected in this region (4, 11–14). However, there is still little information on distribution of HBV or HAV genotypes among febrile and jaundiced patients in Mombasa County. Some studies have been done in this region but among high-risk populations of intravenous drug users (IDUs). Screening for disease burden have also been evaluated but not among the high-risk populations like IDUs. Therefore, this study was conducted in order to determine the prevalence and genetic diversity of HAV and HBV among patients presenting with jaundice and other symptoms at Coast General Hospital in Mombasa County, Kenya.

## Methods

A hospital based cross-sectional study was done and a total of 222 samples collected among febrile and jaundiced patients seeking medical care at the casualty, paediatric clinic, hepatic clinic of out and in-patient department seeking medical services at Coast General Hospital, Mombasa County, Kenya. The study participants, both in and outpatients, were recruited during the period between February 2018 and August 2018. Ethical approval was obtained from the Kenyatta University Ethical Review Committee and National Ethical Review Committee before execution. Purposive sampling was used to recruit study participants. Size of the sample was determined with an estimated prevalence of 17.3%, using the formula:

$$n=\frac{{\text{Z}}^{2}\;P(1-P)}{d^{2}}$$

Where;

*n* = Sample Size
*Z* = Statistic level of confidence
*P* = Estimated prevalence of HBV (17.3%, 0.05)
*d* = level of precision (5%, d = 0.05)
$n=\frac{1.96^{2}\;0.173\;(1-0.173)}{0.05^{2}}$
*n* = 220 blood sample

The patients consented, a self-structured questionnaires administered and data on age, gender and clinical history of jaundiced patients in relation to HAV and HBV infections and clinical symptoms were collected. Venous blood samples were obtained and screened for HAV and HBV infections.

### Serological Analysis

HAV infections were confirmed by anti-HAV IgM using International Immunodiagnostics anti-HAV Ab ELISA kit (International Immunodiagnostics Inc, Carlifornia, USA) (2, 10) and HBV using hepatitis B surface antigen (HBsAg) by Hepanostika^®^ HBsAg Ultra ELISA kit (Biomerieux, Netherlands) (11, 14). Viral deoxyribonucleic acid (DNA) was extracted from confirmed positive samples.

### Viral DNA Extraction and Amplifications

Approximately 5.0 mL blood samples were used and viral DNA extracted using QIAamp DNA blood mini viral kit (Qiagen GmbH, Hilden, Germany) according to manufacturer’s instructions (12), Hepatitis B virus *pol* (HBV-*pol)* gene was amplified by nested polymerase chain reaction (PCR) using primers F_1_ 5′-CCTGCTGGTGGCTCCAGTTC-3′ and R_1_ 5′-CGTCCCGCG (AC) AGGATCCAGTT-3′ in the first PCR and the second PCR primers were F_2_ 5′-CYTGGCCWAAATTCGCAGTCCC-3′ and R_2_ 5′-GCAAANCCCAAAAGACCACAAT-3′ (12) (Table 1). This was performed in 50 μL reaction volume constituting 20 μL genomic DNA, 10 μL 10x PCR buffer (Qiagen), 4 μL dNTPs (Thermofisher Scientific), 2.5 μL of each primer sequence, 0.5 μL hot start Taq DNA polymerase, (Qiagen GmbH, Hilden, Germany) and 10.5 μL of 2 mM MgCl_2_. The amplifications conditions were similar to hot start, initial activation at 95 °C for 15 min followed by 35 cycles denaturation at 94 °C for 45 s, annealing at 60 °C for 45 s and extension at 72 °C for 60 s, followed by final extension at 72 °C for 10 min were used in both first and second round PCRs (11, 12, 14). The PCR amplification was confirmed by visualisation with ethidium bromide staining of the gel. The confirmed products from second round PCR were then purified using QIAquick kit (Qiagen Inc., Valencia, CA) (11, 14) followed by bidirectional population sequencing using an automated sequencer ABI 377 (Applied Bio systems, Foster City, CA) (12).

### Phylogenetic Analysis

The generated sequences were phylogenetic analysed using MEGA X version 10.0.4. The generated sequences were aligned using CLUSTAL W version 2.1 together with reference sequence from the NCBI GenBank. The rate of occurrence of nucleotide substitution was measured by Kimura-2 parameter model (12, 14) and phylogenetic tree was constructed using neighbour-joining method. The tree was visualised using Tree View Software version 1.6.6 at 1000 bootstrap replicates.

### Statistical Analysis

Age, gender, marital status, occupation and area of residence were compared in relation to HBV infection using 5 × 2 Chi-square. Odds ratio (OR) at 95% confidence interval (CI) for gender and marital status were compared with respect to HBV infection. Tukey’s HSD post-hoc test was used in determining significant differences between sample means of HBV positive infection cases at 95% confidence level between the age groups and gender of patients, respectively. A *P*-value of < 0.05 was considered statistically significant.

## Results

### Demographic Characteristics of the Patients

A total of 222 participants were recruited into the study. Of these, *n* = 124 (55.9%) were females and *n* = 98 (44.1%) were males. Their ages ranged between 4 months old and 75 years old with the mean of 23.6 ± 17.3 years old and a standard deviation of 17.3. For females, mean age was 22.4 ± 15.5 years old with a standard deviation of 15.5. In addition, men had a mean of 25.1 ± 19.2 years old and a standard deviation of 19.2. More than half of the study participants were not married (*n* = 142 [64.0%]) who mostly resided in Likoni (*n* = 92 [41.4%]) followed by Kisauni (*n* = 54 [24.3%]). Most of the study participants were also unemployed (*n* = 182 [82.0%]) (Table 2).

### Prevalence of HAV and HBV

In a total of 222 patients who were screened, no HAV was detected. However, *n* = 47 (21.2%) patients tested positive for HBsAg. This accounted for 21.2% overall HBV prevalence. Hepatitis B prevalence among the unmarried was not significantly higher 23.2% or (OR 1.427; 95% CI: 0.712, 2.862).

We determined if marital status had any influence on HBV infection. Marital status had no influence on HBV infection (OR 1.427; 95% CI: 0.712, 2.862).

From the distribution of infection, area of residence had no predisposing risk factor to HBV infection or significant levels of infection (*P* = 0.670). In contrary, occupation was found to be a risk factor to HBV infection (*P* = 0.002). Those unemployed had significant high levels of participants infected (*P* = 0.002). Prevalence of hepatitis B was higher among males than females with *n* = 39 (39.8 %) and *n* = 8 (6.5 %) prevalence, respectively, (OR 0.104; 95% CI: 0.046, 0.238). Therefore, males had significant higher infections in comparison to females. Across ages, those aged between 25.0 years old and 38.0 years old, 26 (38.2 %) (*P* < 0.022) were the most affected while children ≤ 10 years old were least affected. Age was determined if it was a risk factor to HBV infection. Age was found to be a risk factor to HBV infection with significant variation across ages (*P* < 0.022) (Table 3).

### HBV Genotypes Analysis

**Figure 1:**
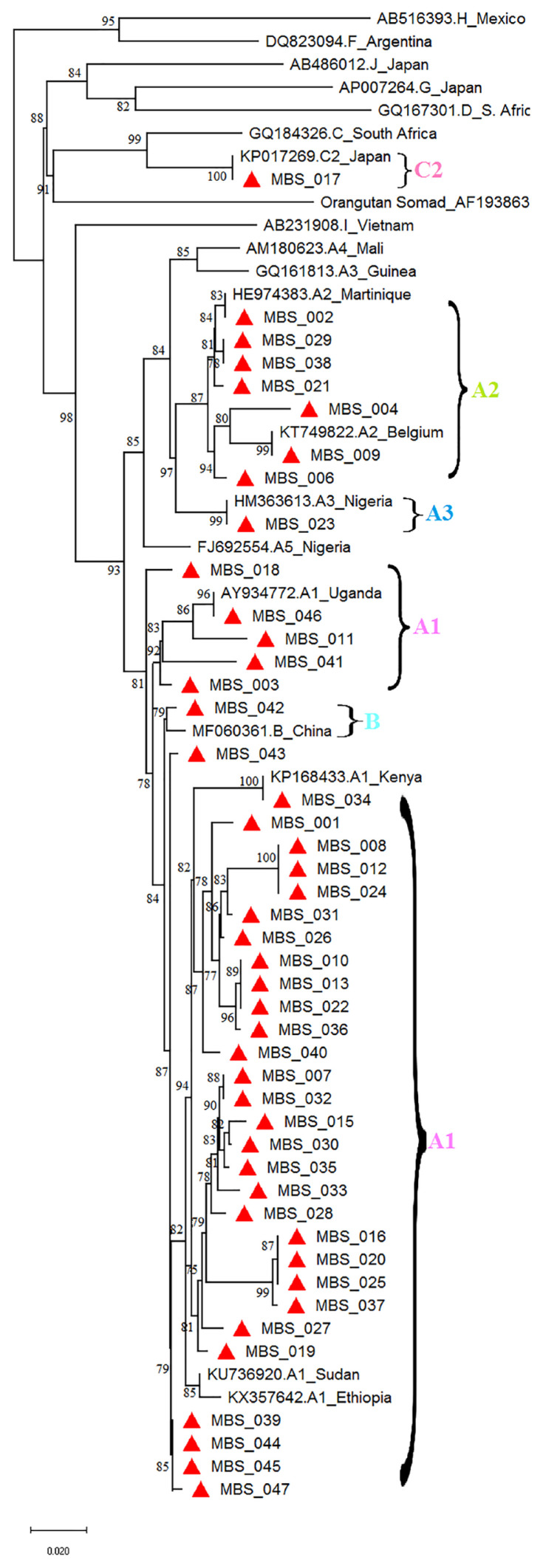
Phylogenetic tree of HBV-pol gene sequences from the Coast General Hospital, Mombasa, Kenya. Neighbour-joining method at 1,000 bootstrap replicates was used. Chimpanzee HBV (Orangutan Samad–AF193863) was the out group used. Bootstrap values > 70% are shown and the HBV isolates from participants of the study are specified in red

Out of 47 sera samples that were analysed, 45 were successfully amplified and sequenced. Generated sequences were phylogenetic analysed using MGEA X version 10.0.4. Phylogenetic analysis revealed that HBV A1 genotype was 35 (74.5%), followed by HBV A2 genotype (*n* = 7 [14.9%]) and *n* = 1 (0.02%) occurrence each for HBV genotypes A3, B and C2 (Figure 1).

## Discussion

In the present study, no HAV was detected. This low HAV prevalence could be linked to the 2016 outbreak where many people were infected and could have resolved their infection to develop long lasting immune responses (IgG) (3). Also, there is likelihood of public awareness following the recent outbreak of HAV infections in the region. This study was conducted immediately after the 2016 sporadic outbreak. This study conforms with previous studies conducted in Ghana [1.3%] (2), Kenya [2%] (9), Kenya [6.3%] (13) and Tanzania [3.1%] (1) that indicate low infection rates due to its sporadic outbreaks associated with poor sanitation and limited access to clean drinking water. In contrary, other studies in the country and other regions revealed high prevalence of 41.7% (15), India [37.25%] (3) and Nigeria [55.2%] (15, 3), all these are being associated with sporadic outbreaks.

Likewise for HBV, the overall prevalence was found to be higher than those previously detected in Kenya [2.36%] (16), [3%] (11), [3.19%] (10), [3.8%] (10), [6.0%] (17), [7.25%] (12), [13.3%] (18), [14.6%] (6, 19)]; Uganda [14.9%] (20) and in other African countries such as Zambia [9.9%]; Malawi [6.7%]; Uganda [4.9%]; Ethiopia [4.7%] and Rwanda [2.4%] (17). This high HBV infection rate in this study is attributed to high likelihood of study participants being of HBV high risk groups especially IDUs, therefore; treatment and management among this group is of necessity (10). In comparison to other studies from other regions and countries, the finding was lower than those of some studies conducted in Ghana [54.2%] (8) and Kenya [50.6%] (13). The variations depicted in the prevalence rates were related to the sample size and the study populations.

Males were significantly highly infected than females (OR 0.104; 95% CI: 0.046, 0.238). This finding confirms previous studies that have been conducted in Kenya (11, 17). This observation could be associated with the fact that most men tend to have multiple sexual partners and possibly engaging in unprotected sex (12). In addition, indulging in drugs and alcoholism could be predisposing factors (11). A high level of infection was observed in the age group 25.0–38.0 years old across gender (*P* < 0.022) followed by age group 39.0–52.0 years (17). This could be due to a strong association of this age group to active sexual age, frequency of sex engagements and sexual experience. The observed finding was similar to other reports from some studies carried out in Ethiopia [4.0%] (21), China [7.21%] (22), Venezuela [8.6%] (23), Nigeria [14.3%] (24) and Togo [26.3%] (25), therefore, affirming the age group as the vulnerable age group to HBV infections. On the contrary, this result was different from an observed finding from a study carried out in Kenya [2.36%] (16), [7.59%] (26) and Tanzania [25.4%] (1, 5). This variation could be associated with varied study design, sample size and study population. On the other hand, the elderly had no HBV infection. This could be associated with their less exposure to HBV high risk behaviours such as indulgence in unprotected sex, alcoholism and drug use (17). Also, the elderly is of sexual inactive age thus less predisposed to HBV infections.

Occupation was determined to be a risk factor in HBV infection since unemployed study participants were reported to have statistically significant higher prevalence (*P* = 0.002). This higher prevalence could be attributed to engagement in high-risk behaviours with an aim of obtaining cash for a living such as commercial sex, multiple sexual partners and intravenous drug use (11). This was similar to findings of a study in Ethiopia daily labourer [12.5%] and students [11.1%] (4, 5, 21) and Eritrea [19.6%] among unemployed women (1). Marital status and area of residence were found not to be the predisposing factors to HBV infection. However, HBV infection among singles was not statistically significant higher, this could be attributed to high tendency of single persons having multiple sexual partners and engaging unprotected sex. This finding is similar to a previous study conducted in Ethiopia (21) and Mexico (19).

The detected HBV sub-genotypes concur with previous studies that have also shown the same distribution (12–14). The phylogenetic relationship revealed that A1 genotype was the most predominantly circulating among the studied subjects. These findings concur with previous studies that have been conducted in the country and other surrounding countries (4, 12–14, 27). HBV A1 sequences clustered with reference sequences from Kenya and Uganda (27). This trend indicates possibility of its East African origin with an implication of these strains circulating locally among Kenyan residents. By virtue of population migration across East African countries could explain this alignment. Sub-genotype A2 sequences aligned with those from Martinique and Belgium as an indication of its possible origins (28, 27). In addition, sequences of sub-genotypes A3, B and genotype C2 clustered with references sequences from Nigeria, China and Japan (7, 27). The detected viral genotypes confirm the persistent and stability of this viral strains circulating within the country. Contrary to previous studies conducted in the country, this study confirms possible existing of other HBV genotypes circulating among Kenyan population. HBV A3, B and C2 genotypes were detected, even though at low proportion. Despite low proportion of the sample used, there are newly detected genotypes in this study. As a result, there is a likelihood of existence of possible other genotypes in this region (4, 12, 14). The D and E genotypes in the previous studies were detected among patients who had history of intravenous drug use (4, 12, 14).

## Conclusion

Despite the fact that HAV occurs sporadically leading to outbreaks, no HAV was detected from the sampled population. However, for HBV, the general prevalence of HBV was found to be *n* = 47 [21.2%] with males and age group 25.0–38.0 years old being the most affected. In addition, from the detected HBV infections, phylogenetic analysis, revealed HBV genotypes A1 as the most predominant (*n* = 35 [74.5%]) followed by sub-genotype A2 (*n* = 7 [14.9%]) with newly detected HBV genotypes A3, B and C2 (*n* = 1 [0.02%]) in this region. Detection of new HBV genotypes in Kenya calls for continuous surveillance of HBV infections and circulating trends of HBV genotypes.

## Figures and Tables

**Table 1:** Primer sequences used in the amplification of HBV-*pol*

Primers	Sequence (5′ 3′)	Base position	Polarity	Reference
F1	CCTGCTGGTGGCTCCAGTTC	nt 56–76	sense	(12)
R1	CGTCCCGCG (AC)AGGATCCAGTT	nt 1395–1416	antisense	(12)
F2	CYTGGCCWAAATTCGCAGTCCC	nt 298 320	sense	(12)
R2	GCAAANCCCAAAAGACCACAAT	nt 997–1019	antisense	(12)

**Table 2:** Demographic characteristics of jaundiced patients at the Coast General Hospital

Gender	*N* = 222	Female *n* (124)	Male *n* (98)	*P*-value
Mean age	23.6±17.3	22.4±15.5	25.1±19.2	
SD	17.3	15.5	19.2	
Age groups				
≤10.0	68	35 (51.5)	33 (48.5)	
11.0–24.0	42	31 (73.8)	11 (26.2)	
25.0 38.0	68	39 (57.4)	29 (42.6)	*P* < 0.022
39.0–52.0	28	11 (39.3)	17 (60.7)	
53.0+	15	6 (40.0)	9 (60.0)	
Marital status				
Single	142 (64.0)	78 (54.9)	64 (45.1)	
Married	80 (36.0)	47 (58.8)	33 (41.2)	
Area of residence				
Likoni	92 (41.4)	50 (54.3)	42 (45.7)	
Kisauni	54 (24.3)	26 (48.1)	28 (51.9)	
Mvita	41 (18.5)	24 (58.5)	17 (41.5)	*P* = 0.067
Mwishomoroni	34 (15.3)	13 (38.2)	21 (61.8)	
Nyali	1 (0.005)	1 (100)	0 (100)	
Occupation				
Unemployed	182 (82.0)	85 (46.7)	96 (52.7)	
Government employed	21 (9.5)	12 (57.1)	9 (42.9)	
Self-employed	19 (8.6)	8 (42.1)	11 (57.9)	*P* = 0.002
Private sector employed	11 (5.0)	5 (45.5)	6 (54.5)	
Housewife	37 (16.7)	14 (37.8)	23 (62.2)	

Notes: 5 × 2 Chi-square was used in to compare the age, area of residence and occupation in relation to HBV infection. *N* represents study population while *n* denotes sample

**Table 3:** Prevalence of HBV by age and gender among jaundiced patients at the Coast General Hospital

Variables		HBsAg		*P*-value	OR

		Positive *f* (%)	Negative *f* (%)		
Age group (years old)	≤10.0 (*N* = 68)	1 (0.01)	67 (98.5)		
	11.0–24.0 (*N* = 42)	10 (23.8)	32 (76.2)		
	25.0 38.0 (*N* = 68)	26 (38.2)	42 (61.8)	*P* < 0.022	
	39.0–52.0 (*N* = 28)	9 (32.1)	19 (67.9)		
	53.0 + (*N* = 15)	1 (6.7)	14 (93.3)		
Sex	Male (*N* = 98)	39 (39.8)	59 (60.2)		
	Female (*N* = 124)	8 (6.5)	116 (93.5)		0.104; 95% CI: 0.046, 0.238
HBV positive (M+F)		47 (21.2)			
Marital status	Single (*N* = 142)	33 (23.2)	109 (76.8)		1.427; 95% CI: 0.712, 2.862
	Married (*N* = 80)	14 (17.5)	66 (82.5)		
Area of residence	Likoni (*N* = 92)	19 (20.7)	73 (79.3)		
	Kisauni (*N* = 54)	15 (27.8)	39 (72.2)		
	Mvita (*N* = 41)	7 (17.1)	34 (82.9)	*P* = 0.670	
	Mwishomoroni (*N* = 34)	6 (17.6)	28 (82.4)		
	Nyali (*N* = 1)	0 (0.0)	1 (100.0)		
Occupation	Unemployed (*N* = 134)	34 (25.4)	100 (74.6)		
	Government employed (*N* = 21)	2 (9.5)	19 (90.5)		
	Self-employed (*N* = 19)	1 (5.3)	18 (94.7)	*P* = 0.002	
	Private sector employed (*N* = 11)	1 (9.1)	10 (90.9)		
	Housewives (*N* = 37)	9 (24.3)	28 (75.7)		

Notes: 5 × 2 Chi-square was used to compare age, area of residence and occupation in relation to HBV infection while OR was used to analyse marital status and sex in relation to HBV infection.
